# 4-Ethyl­anilinium 2-carb­oxy­acetate

**DOI:** 10.1107/S1600536810029648

**Published:** 2010-07-31

**Authors:** De-Hong Wu, Qi-Qi Wu

**Affiliations:** a College of Chemistry and Chemical Engineering, Southeast University, Nanjing 210096, People’s Republic of China

## Abstract

In the crystal structure of the title compound, C_8_H_12_N^+^·C_3_H_3_O_4_^−^, the hydrogen malonate anions are linked into infinite chains parallel to the *b* axis by inter­molecular O—H⋯O hydrogen bonds of the type COO^−^⋯HO_2_C in a head-to-tail fashion. The 4-ethyl­anilinium cations link adjacent anion chains by inter­molecular N—H⋯O hydrogen bonds into a two-dimensional network parallel to the *b* and *c* axes.

## Related literature

For background to mol­ecular–ionic compounds, see: Czupiński *et al.* (2002 ▶); Katrusiak & Szafrański (2006 ▶); Chen (2009 ▶); Wang (2010 ▶).
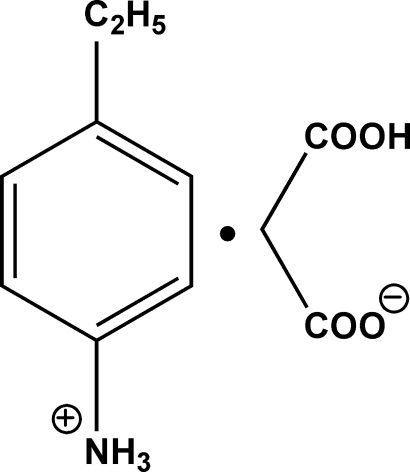


## Experimental

### 

#### Crystal data

C_8_H_12_N^+^·C_3_H_3_O_4_^−^
*M* *_r_* = 225.24
Monoclinic, 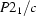

*a* = 13.439 (3) Å
*b* = 9.2914 (19) Å
*c* = 8.8827 (18) Å
β = 99.177 (10)°
*V* = 1095.0 (4) Å^3^
*Z* = 4
Mo *K*α radiation
μ = 0.10 mm^−1^
*T* = 291 K
0.36 × 0.32 × 0.28 mm

#### Data collection

Rigaku SCXmini diffractometer
Absorption correction: multi-scan (*CrystalClear*; Rigaku, 2005 ▶) *T*_min_ = 0.963, *T*_max_ = 0.971
11013 measured reflections
2510 independent reflections
1995 reflections with *I* > 2σ(*I*)
*R*_int_ = 0.042

#### Refinement

*R*[*F* ^2^ > 2σ(*F* ^2^)] = 0.060
*wR*(*F* ^2^) = 0.161
*S* = 1.05
2510 reflections
147 parameters
H-atom parameters constrained
Δρ_max_ = 0.42 e Å^−3^
Δρ_min_ = −0.43 e Å^−3^

### 

Data collection: *CrystalClear* (Rigaku, 2005 ▶); cell refinement: *CrystalClear*; data reduction: *CrystalClear*; program(s) used to solve structure: *SHELXS97* (Sheldrick, 2008 ▶); program(s) used to refine structure: *SHELXL97* (Sheldrick, 2008 ▶); molecular graphics: *SHELXTL* (Sheldrick, 2008 ▶); software used to prepare material for publication: *SHELXTL*.

## Supplementary Material

Crystal structure: contains datablocks I, global. DOI: 10.1107/S1600536810029648/kj2151sup1.cif

Structure factors: contains datablocks I. DOI: 10.1107/S1600536810029648/kj2151Isup2.hkl

Additional supplementary materials:  crystallographic information; 3D view; checkCIF report

## Figures and Tables

**Table 1:** Hydrogen-bond geometry (Å, °)

*D*—H⋯*A*	*D*—H	H⋯*A*	*D*⋯*A*	*D*—H⋯*A*
N1—H1*A*⋯O1	0.89	2.08	2.777 (2)	134
N1—H1*B*⋯O1^i^	0.89	2.57	3.200 (3)	129
N1—H1*B*⋯O2^ii^	0.89	2.27	2.930 (2)	131
N1—H1*C*⋯O3^iii^	0.89	2.31	2.815 (2)	116
N1—H1*A*⋯O4^ii^	0.89	2.28	2.885 (2)	125
O4—H4⋯O2^iv^	0.91	1.64	2.532 (2)	167

Symmetry codes: (i) 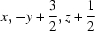; (ii) 
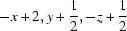
; (iii) 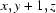; (iv) 
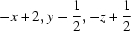
.

**Fig. 1.**
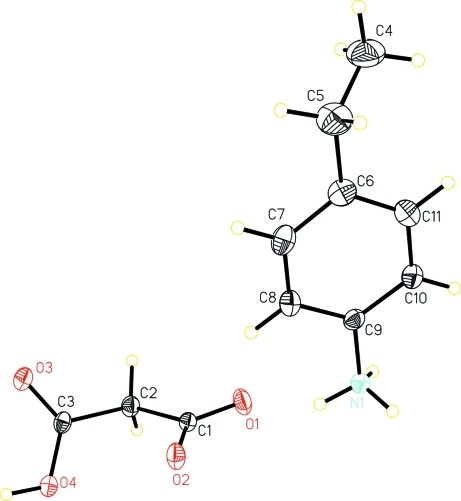
The molecular structure of the title compound, showing the atomic numbering scheme with 30% probability displacement ellipsoids.

**Fig. 2.**
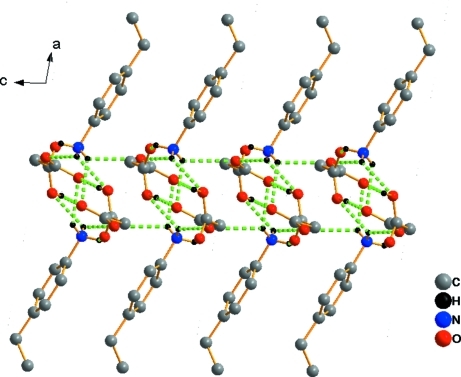
A view of the packing of the title compound, stacking along the b axis. Dashed lines indicate hydrogen bonds.

## supplementary crystallographic information

### Comment

Recently much attention has been devoted to simple molecular–ionic crystals containing organic cations and anions due to the tunability of their special structural features and their interesting physical properties (Czupiński *et al.*, 2002; Katrusiak & Szafrański, 2006). For similar structures, see: Chen, 2009; Wang, 2010. The title compound has been synthesized in our laboratory and its crystal structure is reported here.

The asymmetric unit of the title compound consists of one 4-ethylanilinium cation and one hydrogen malonate anion (Fig 1), in which complete transfer of a single H atom from the acid component to the basic component has occurred. In the crystal structure, the hydrogen malonate anions are linked into one-dimensional infinite chains parallel to *b*-axis by intermolecular O—H···O hydrogen bonds of the type COO^-^···HO_2_C in a "head-to-tail" fashion. The 4-ethylanilinium cations link adjacent anion chains by intermolecular N—H···O hydrogen bonds into a two-dimensional network running parallel to the *b* and *c*-axes .(Fig 2). Hydrogen bonds of intermolecular N—H···O and O—H···O make great contribution to the stability of the crystal structure (Table 1).

### Experimental

1.04 g (10 mmol) malonic acid hydrate was dissolved in 50 ml ethanol, to which 1.21 g (10 mmol) 4-ethybenzenamine was added to afford a solution without any precipitation under stirring at ambient temperature. Single crystals suitable for X-ray structure analysis were obtained by the slow evaporation of the above solution after 3 days in air.

The dielectric constant of the compound as a function of temperature indicates that the permittivity is basically temperature-independent (*ε* = C/(T–T_0_)), suggesting that this compound is not ferroelectric or there may be no distinct phase transition occurring within the measured temperature range between 93 K and 362 K (m.p. 99 *^o^*C).

### Refinement

H atoms except for H4 were placed in calculated positions (N—H = 0.89 Å; C—H = 0.93Å for C*sp^2^* atoms and C—H = 0.96Å and 0.97Å for C*sp^3^* atoms), assigned fixed *U*_iso_ values [*U*_iso_ = 1.2*U*eq(C*sp^2^*) and 1.5*U*eq(C*sp^3^*,*N*)] and allowed to ride. The H4 atom bonding with O4 was found with O—H bond distance of 0.9084Åin the difference electron density map.

### Crystal data

**Table:**

C_8_H_12_N^+^·C_3_H_3_O_4_^−^	*F*(000) = 480
*M**_r_* = 225.24	*D*_x_ = 1.366 Mg m^−^^3^
Monoclinic, *P*2_1_/*c*	Mo *K*α radiation, λ = 0.71073 Å
Hall symbol: -P 2ybc	Cell parameters from 9421 reflections
*a* = 13.439 (3) Å	θ = 3.1–27.6°
*b* = 9.2914 (19) Å	µ = 0.10 mm^−^^1^
*c* = 8.8827 (18) Å	*T* = 291 K
β = 99.177 (10)°	Block, colorless
*V* = 1095.0 (4) Å^3^	0.36 × 0.32 × 0.28 mm
*Z* = 4	

### Data collection

**Table:**

Rigaku SCXmini diffractometer	2510 independent reflections
Radiation source: fine-focus sealed tube	1995 reflections with *I* > 2σ(*I*)
graphite	*R*_int_ = 0.042
Detector resolution: 13.6612 pixels mm^-1^	θ_max_ = 27.5°, θ_min_ = 3.1°
ω scans	*h* = −17→17
Absorption correction: multi-scan (*CrystalClear*; Rigaku, 2005)	*k* = −12→12
*T*_min_ = 0.963, *T*_max_ = 0.971	*l* = −11→11
11013 measured reflections	

### Refinement

**Table:**

Refinement on *F*^2^	Secondary atom site location: difference Fourier map
Least-squares matrix: full	Hydrogen site location: inferred from neighbouring sites
*R*[*F*^2^ > 2σ(*F*^2^)] = 0.060	H-atom parameters constrained
*wR*(*F*^2^) = 0.161	*w* = 1/[σ^2^(*F*_o_^2^) + (0.0682*P*)^2^ + 0.8364*P*] where *P* = (*F*_o_^2^ + 2*F*_c_^2^)/3
*S* = 1.05	(Δ/σ)_max_ < 0.001
2510 reflections	Δρ_max_ = 0.42 e Å^−^^3^
147 parameters	Δρ_min_ = −0.43 e Å^−^^3^
0 restraints	Extinction correction: *SHELXTL* (Sheldrick, 2008), Fc^*^=kFc[1+0.001xFc^2^λ^3^/sin(2θ)]^-1/4^
Primary atom site location: structure-invariant direct methods	Extinction coefficient: 0.038 (5)

### Special details

**Table:**

Geometry. All e.s.d.'s (except the e.s.d. in the dihedral angle between two l.s. planes) are estimated using the full covariance matrix. The cell e.s.d.'s are taken into account individually in the estimation of e.s.d.'s in distances, angles and torsion angles; correlations between e.s.d.'s in cell parameters are only used when they are defined by crystal symmetry. An approximate (isotropic) treatment of cell e.s.d.'s is used for estimating e.s.d.'s involving l.s. planes.
Refinement. Refinement of *F*^2^ against ALL reflections. The weighted *R*-factor *wR* and goodness of fit *S* are based on *F*^2^, conventional *R*-factors *R* are based on *F*, with *F* set to zero for negative *F*^2^. The threshold expression of *F*^2^ > σ(*F*^2^) is used only for calculating *R*-factors(gt) *etc*. and is not relevant to the choice of reflections for refinement. *R*-factors based on *F*^2^ are statistically about twice as large as those based on *F*, and *R*- factors based on ALL data will be even larger.

### Fractional atomic coordinates and isotropic or equivalent isotropic displacement parameters (Å^2^)

**Table:**

	*x*	*y*	*z*	*U*_iso_*/*U*_eq_	
C1	0.90859 (14)	0.4455 (2)	0.0928 (2)	0.0260 (4)	
C2	0.89084 (16)	0.3160 (2)	−0.0141 (2)	0.0290 (5)	
H2A	0.8213	0.3177	−0.0645	0.035*	
H2B	0.9332	0.3261	−0.0923	0.035*	
C3	0.91163 (15)	0.1717 (2)	0.0613 (2)	0.0265 (4)	
C4	0.3837 (2)	0.7987 (4)	0.3598 (4)	0.0718 (10)	
H4A	0.3281	0.7797	0.4124	0.108*	
H4B	0.3704	0.7573	0.2595	0.108*	
H4C	0.3926	0.9008	0.3517	0.108*	
C5	0.4769 (2)	0.7341 (4)	0.4458 (3)	0.0605 (8)	
H5A	0.4657	0.6316	0.4555	0.073*	
H5B	0.4877	0.7745	0.5478	0.073*	
C6	0.57213 (18)	0.7548 (3)	0.3778 (3)	0.0405 (6)	
C7	0.63668 (19)	0.6409 (3)	0.3677 (3)	0.0442 (6)	
H7A	0.6205	0.5508	0.4025	0.053*	
C8	0.72484 (18)	0.6569 (2)	0.3073 (3)	0.0388 (5)	
H8A	0.7669	0.5785	0.3006	0.047*	
C9	0.74915 (15)	0.7901 (2)	0.2574 (2)	0.0300 (5)	
C10	0.68750 (17)	0.9066 (2)	0.2664 (3)	0.0388 (5)	
H10A	0.7046	0.9967	0.2328	0.047*	
C11	0.59904 (19)	0.8877 (3)	0.3267 (3)	0.0453 (6)	
H11A	0.5570	0.9662	0.3327	0.054*	
N1	0.84388 (13)	0.80909 (19)	0.1986 (2)	0.0347 (5)	
H1A	0.8755	0.7249	0.1992	0.052*	
H1B	0.8828	0.8714	0.2571	0.052*	
H1C	0.8311	0.8426	0.1036	0.052*	
O1	0.88090 (13)	0.56380 (16)	0.03767 (19)	0.0412 (4)	
O2	0.95091 (12)	0.42353 (15)	0.22859 (17)	0.0352 (4)	
O3	0.84347 (12)	0.08947 (16)	0.0783 (2)	0.0413 (4)	
O4	1.00669 (11)	0.14348 (15)	0.10516 (18)	0.0337 (4)	
H4	1.0153	0.0579	0.1549	0.105 (14)*	

### Atomic displacement parameters (Å^2^)

**Table:**

	*U*^11^	*U*^22^	*U*^33^	*U*^12^	*U*^13^	*U*^23^
C1	0.0251 (9)	0.0210 (9)	0.0323 (10)	−0.0010 (7)	0.0058 (8)	0.0015 (7)
C2	0.0335 (11)	0.0246 (10)	0.0277 (10)	−0.0018 (8)	0.0011 (8)	0.0019 (8)
C3	0.0333 (11)	0.0210 (9)	0.0261 (10)	−0.0025 (8)	0.0072 (8)	−0.0048 (7)
C4	0.0498 (17)	0.083 (2)	0.088 (2)	−0.0032 (16)	0.0301 (17)	0.0124 (19)
C5	0.0609 (18)	0.071 (2)	0.0559 (17)	0.0003 (15)	0.0279 (14)	0.0118 (15)
C6	0.0422 (13)	0.0461 (14)	0.0340 (11)	−0.0037 (10)	0.0088 (10)	0.0026 (10)
C7	0.0531 (15)	0.0344 (12)	0.0463 (14)	−0.0079 (11)	0.0117 (11)	0.0074 (10)
C8	0.0417 (13)	0.0267 (11)	0.0481 (13)	−0.0004 (9)	0.0074 (10)	0.0040 (9)
C9	0.0277 (10)	0.0285 (10)	0.0316 (10)	−0.0039 (8)	−0.0019 (8)	−0.0002 (8)
C10	0.0370 (12)	0.0269 (11)	0.0514 (14)	−0.0021 (9)	0.0037 (10)	0.0037 (9)
C11	0.0422 (13)	0.0398 (13)	0.0543 (15)	0.0065 (10)	0.0091 (11)	0.0003 (11)
N1	0.0280 (9)	0.0244 (9)	0.0497 (11)	−0.0017 (7)	0.0003 (8)	0.0051 (8)
O1	0.0511 (10)	0.0242 (8)	0.0454 (9)	0.0081 (7)	−0.0010 (7)	0.0041 (7)
O2	0.0499 (9)	0.0225 (7)	0.0305 (8)	0.0008 (6)	−0.0021 (7)	−0.0014 (6)
O3	0.0363 (9)	0.0264 (8)	0.0626 (11)	−0.0054 (6)	0.0118 (8)	0.0058 (7)
O4	0.0328 (8)	0.0222 (7)	0.0445 (9)	−0.0023 (6)	0.0010 (6)	0.0024 (6)

### Geometric parameters (Å, °)

**Table:**

C1—O1	1.237 (2)	C6—C7	1.381 (4)
C1—O2	1.265 (2)	C6—C11	1.383 (3)
C1—C2	1.528 (3)	C7—C8	1.384 (3)
C2—C3	1.505 (3)	C7—H7A	0.9300
C2—H2A	0.9700	C8—C9	1.372 (3)
C2—H2B	0.9700	C8—H8A	0.9300
C3—O3	1.220 (2)	C9—C10	1.373 (3)
C3—O4	1.301 (2)	C9—N1	1.462 (3)
C4—C5	1.485 (5)	C10—C11	1.390 (3)
C4—H4A	0.9600	C10—H10A	0.9300
C4—H4B	0.9600	C11—H11A	0.9300
C4—H4C	0.9600	N1—H1A	0.8900
C5—C6	1.512 (4)	N1—H1B	0.8900
C5—H5A	0.9700	N1—H1C	0.8900
C5—H5B	0.9700	O4—H4	0.9084
			
O1—C1—O2	125.59 (19)	C7—C6—C5	120.6 (2)
O1—C1—C2	116.51 (18)	C11—C6—C5	121.8 (2)
O2—C1—C2	117.89 (17)	C6—C7—C8	121.9 (2)
C3—C2—C1	115.15 (16)	C6—C7—H7A	119.1
C3—C2—H2A	108.5	C8—C7—H7A	119.1
C1—C2—H2A	108.5	C9—C8—C7	119.0 (2)
C3—C2—H2B	108.5	C9—C8—H8A	120.5
C1—C2—H2B	108.5	C7—C8—H8A	120.5
H2A—C2—H2B	107.5	C8—C9—C10	121.0 (2)
O3—C3—O4	123.86 (19)	C8—C9—N1	119.35 (19)
O3—C3—C2	121.53 (19)	C10—C9—N1	119.61 (19)
O4—C3—C2	114.60 (17)	C9—C10—C11	119.0 (2)
C5—C4—H4A	109.5	C9—C10—H10A	120.5
C5—C4—H4B	109.5	C11—C10—H10A	120.5
H4A—C4—H4B	109.5	C6—C11—C10	121.6 (2)
C5—C4—H4C	109.5	C6—C11—H11A	119.2
H4A—C4—H4C	109.5	C10—C11—H11A	119.2
H4B—C4—H4C	109.5	C9—N1—H1A	109.5
C4—C5—C6	116.2 (2)	C9—N1—H1B	109.5
C4—C5—H5A	108.2	H1A—N1—H1B	109.5
C6—C5—H5A	108.2	C9—N1—H1C	109.5
C4—C5—H5B	108.2	H1A—N1—H1C	109.5
C6—C5—H5B	108.2	H1B—N1—H1C	109.5
H5A—C5—H5B	107.4	C3—O4—H4	111.3
C7—C6—C11	117.6 (2)		
			
O1—C1—C2—C3	−171.71 (18)	C6—C7—C8—C9	0.6 (4)
O2—C1—C2—C3	9.0 (3)	C7—C8—C9—C10	−0.1 (3)
C1—C2—C3—O3	108.1 (2)	C7—C8—C9—N1	177.8 (2)
C1—C2—C3—O4	−72.1 (2)	C8—C9—C10—C11	−0.3 (3)
C4—C5—C6—C7	−134.2 (3)	N1—C9—C10—C11	−178.2 (2)
C4—C5—C6—C11	47.1 (4)	C7—C6—C11—C10	0.3 (4)
C11—C6—C7—C8	−0.7 (4)	C5—C6—C11—C10	179.0 (2)
C5—C6—C7—C8	−179.4 (3)	C9—C10—C11—C6	0.2 (4)

### Hydrogen-bond geometry (Å, °)

**Table:**

*D*—H···*A*	*D*—H	H···*A*	*D*···*A*	*D*—H···*A*
N1—H1A···O1	0.89	2.08	2.777 (2)	134
N1—H1B···O1^i^	0.89	2.57	3.200 (3)	129
N1—H1B···O2^ii^	0.89	2.27	2.930 (2)	131
N1—H1C···O3^iii^	0.89	2.31	2.815 (2)	116
N1—H1A···O4^ii^	0.89	2.28	2.885 (2)	125
O4—H4···O2^iv^	0.91	1.64	2.532 (2)	167

Symmetry codes: (i) *x*, −*y*+3/2, *z*+1/2; (ii) −*x*+2, *y*+1/2, −*z*+1/2; (iii) *x*, *y*+1, *z*; (iv) −*x*+2, *y*−1/2, −*z*+1/2.
